# *Colletotrichum* spp. from Soybean Cause Disease on Lupin and Can Induce Plant Growth-Promoting Effects

**DOI:** 10.3390/microorganisms9061130

**Published:** 2021-05-24

**Authors:** Louisa Wirtz, Nelson Sidnei Massola Júnior, Renata Rebellato Linhares de Castro, Brigitte Ruge-Wehling, Ulrich Schaffrath, Marco Loehrer

**Affiliations:** 1 Department of Plant Physiology, RWTH Aachen University, 52056 Aachen, Germany; louisa.wirtz@rwth-aachen.de (L.W.); schaffrath@bio3.rwth-aachen.de (U.S.); 2 Department of Plant Pathology and Nematology, ESALQ, University of São Paulo, Piracicaba 13418-900, SP, Brazil; nmassola@usp.br (N.S.M.J.); renatarebellatolinhares@hotmail.com (R.R.L.d.C.); 3 Institute for Breeding Research on Agricultural Crops, Julius Kühn-Institut, 18190 Groß Lüsewitz, Germany; brigitte.ruge-wehling@julius-kuehn.de

**Keywords:** *Colletotrichum*, anthracnose, soybean, lupin, protein crop plant, commensalism, mutualism, parasitism, plant growth-promoting effect

## Abstract

Protein crop plants such as soybean and lupin are attracting increasing attention because of their potential use as forage, green manure, or for the production of oil and protein for human consumption. Whereas soybean production only recently gained more importance in Germany and within the whole EU in frame of protein strategies, lupin production is already well-established in Germany. The cultivation of lupins is impeded by the hemibiotrophic ascomycete *Colletotrichum lupini*, the causal agent of anthracnose disease. Worldwide, soybean is also a host for a variety of *Colletotrichum* species, but so far, this seems to not be the case in Germany. Cross-virulence between lupin- and soybean-infecting isolates is a potential threat, especially considering the overlap of possible soybean and lupin growing areas in Germany. To address this question, we systematically investigated the interaction of different *Colletotrichum* species isolated from soybean in Brazil on German soybean and lupin plant cultivars. Conversely, we tested the interaction of a German field isolate of *C. lupini* with soybean. Under controlled conditions, *Colletotrichum* species from soybean and lupin were able to cross-infect the other host plant with varying degrees of virulence, thus underpinning the potential risk of increased anthracnose diseases in the future. Interestingly, we observed a pronounced plant growth-promoting effect for some host–pathogen combinations, which might open the route to the use of beneficial biological agents in lupin and soybean production.

## 1. Introduction

In view of the steadily growing world population and the increasing demand for sustainable food production, it has become clear that dietary protein cannot be provided through animal products alone. Soybean is the primary source for non-animal protein and the most cultivated protein crop plant on a global scale. In Europe, soybean and soybean-derived products are imported for the most part [1]. Because European consumers are increasingly shifting their purchase behavior to non-GMO products from sustainable production, soybean imports from abroad are under debate. Hence, the independent production of protein crop plants in Germany becomes increasingly attractive, and lupin represents an appealing alternative to soybean [1,2].

Currently, soybean cultivation in Germany is mostly affected by the fungal disease *Sclerotinia sclerotiorum* causing stem rot (white mold) and the disease complex *Diaporthe* spp./*Phomopsis* spp., causing seed and stem blight [3,4,5]. As *Colletotrichum* is a significant problem in worldwide soybean production and because of increasing production, it is only a matter of time until anthracnose will become a severe problem on soybean in Germany. Recently, the causal agents of anthracnose on soybean were systematically investigated in Brazil, describing *C. truncatum* as the major anthracnose-causing species, but also identifying *C. plurivorum* as a novel species capable of causing [6,7,8,9,10].

In past decades, lupin production has been negatively affected by the hemibiotrophic ascomycete *Colletotrichum lupini*, causing anthracnose disease [2,11,12]. The influence of anthracnose epidemics was severe, partially due to focusing on breeding low-alkaloid-containing white lupin in the past, which is especially susceptible to *C. lupini* [13]. Breeding for resistance of lupin against *C. lupini* is slowly progressing, but the impact of the disease on production success is still severe and alternative plant protection methods are needed [14,15]

In this study, we performed inoculation experiments with *Colletotrichum* species isolated from soybean and lupin in Brazil and Germany, respectively, on soybean cultivar Abelina and lupin cultivars Amiga and Lila Baer, used in Germany to evaluate the risk of cross-infection in a scenario of increased legume production. The results from this study shall contribute to planning of current and future plant breeding goals and open the road to novel plant protection strategies.

## 2. Materials and Methods

### 2.1. Plant Material and Growth Conditions

Seeds of *Glycine max* cv. Abelina (not inoculated with rhizobia) and *Lupinus angustifolius* cv. Lila Baer were kindly provided by I.G. Pflanzenzucht GmbH, Munich, Germany. Seeds of *Lupinus albus* cv. Amiga were obtained from Revierberatung Wolmersdorf GmbH & Co. KG, Wolmersdorf, Germany. Plants were grown in a climate chamber (day/night cycle: 16 h light, 350 µmol m^−2^ s^−1^ PAR, 24 °C, 65% RH; 8 h dark, 20 °C, 80% RH) in ED73 substrate (Balster Einheitserdewerk GmbH, Fröndenberg, Germany).

### 2.2. Fungal Isolates and Culture Conditions

The German field isolate of *Colletotrichum lupini* BBA70358 (= CBS 109222) was deposited at the CBS collection of the Westerdijk Fungal Biodiversity Institute (Utrecht, The Netherlands) and was donated to B. Ruge-Wehling by H. I. Nirenberg. *C. truncatum* isolates (Table 1) were provided by N.S. Massola Jr. and stored at the culture collection of the Department of Plant Pathology and Nematology, ESALQ, University of São Paulo, Brazil [8]. Isolates of *C. plurivorum* (Table 1) were also provided by N.S. Massola Jr. and stored at the same culture collection [7]. Richard O’Connell (BIOGER, INRA, Paris, France) kindly provided *C. higginsianum* IMI 349063. Isolate names in brackets refer to alternative names from either the Westerdijk Institute culture collection (https://wi.knaw.nl/page/Collection, accessed 1 May 2021) or the Universidade Federal de Pernambuco fungal culture collection (http://www.wfcc.info/ccinfo/index.php/collection/by_id/604, accessed 1 May 2021). All fungi were propagated on potato extract glucose agar (PDA) medium (Carl Roth GmbH + Co. KG, Karlsruhe, Germany). The fungal cultures were incubated at 25 °C with a 12 h day/night cycle under cool white fluorescent lamps. Every 7–10 days, fungal isolates were transferred to fresh PDA plates. To prevent degeneration of isolates (i.e., reduced sporulation), at regular intervals (every three months), material from stocks was used to start a new culture.

### 2.3. Hypocotyl Assay

The protocol for the toothpick inoculation assay was adapted from Scandiani et al. (2011) [18]. To prepare hypocotyls of soybean and lupin, seeds were placed on wet filter paper and incubated in the dark. Seeds that germinated after 2–3 days were planted in ED73 soil and placed in a climate chamber as described above. The hypocotyls were ready for the assay at the plant growth stage of formation of the first leaf. Sterile toothpick tips (1.0–1.5 cm) were placed around a growing fungal colony on PDA plates and used for hypocotyl inoculation when fully overgrown by fungal mycelium. The inoculated toothpick tips were pushed into hypocotyls using sterile forceps. After seven days, lesion length was measured using a Leica MZ125 stereomicroscope equipped with a digital JVC KYF 750 camera using Diskus software (Technisches Büro Hilgers, Köngiswinter, Germany).

### 2.4. Seed Inoculation Assay

Soybean and lupin seeds were surface sterilized by washing in 70% EtOH for 1′, followed by 3× rinsing in sterile distilled water. Afterwards, the seeds were washed in 1% sodium hypochlorite solution again followed by 3× rinsing in sterile distilled water. Seeds were dried under sterile conditions for 24 h. For this assay, PDA-mannitol agar plates, adjusted to an osmotic potential of 1 MPa by adding 74.69 g L^−1^ D-mannitol, were prepared following the method described in Scandiani et al. (2011) [18]. Fungal cultures were grown on PDA-mannitol plates until the colonies spread all over the plates. Surface-sterilized seeds were placed on those plates and incubated for 48 h under the growth conditions described for fungal cultures above. The inoculated seeds were planted in ED73 substrate, and seedling growth was monitored. The mock control consisted of surface-sterilized seeds, that were incubated on PDA-mannitol plates without fungus. Plant development was rated in the following categories: 0—seedling not emerged; 1—seedling development arrested after emergence; 2—slower or impaired seedling development in comparison to seedlings emerging from mock-inoculated seeds; 3 seedling development like mock; 4—faster/better development in comparison to mock. At later growth stages, either plant height was measured or a scoring according to a lupin-adapted BBCH development scale was applied [19,20].

### 2.5. Statistical Evaluation

SigmaStat (Systat Software Inc., San Jose, CA, USA) was used for statistical analysis. The toothpick-inoculation experiments were evaluated in comparison with mock control by a one-way ANOVA analysis on ranks, followed by a Dunn’s post hoc test (*p* < 0.05). The seed inoculation experiments produced either nominal data (height measurements) or ordinal data (scoring scale). For ordinal data, median and mean absolute deviation from the median are depicted. Statistical significance in relation to mock was tested with a one-way ANOVA analysis followed by Dunnett’s (*p* < 0.05, number sign) or Holm-Sidak (*p* < 0.05, asterisk) post hoc test. For growth evaluation assays based on BBCH scale, the test for statistical significance was performed by a one-way ANOVA analysis on ranks, followed by Dunn’s post hoc test (*p* < 0.05, asterisk).

## 3. Results

### 3.1. Cross-Infection Assays on Soybean and Lupin Hypocotyl

**Figure 1:**
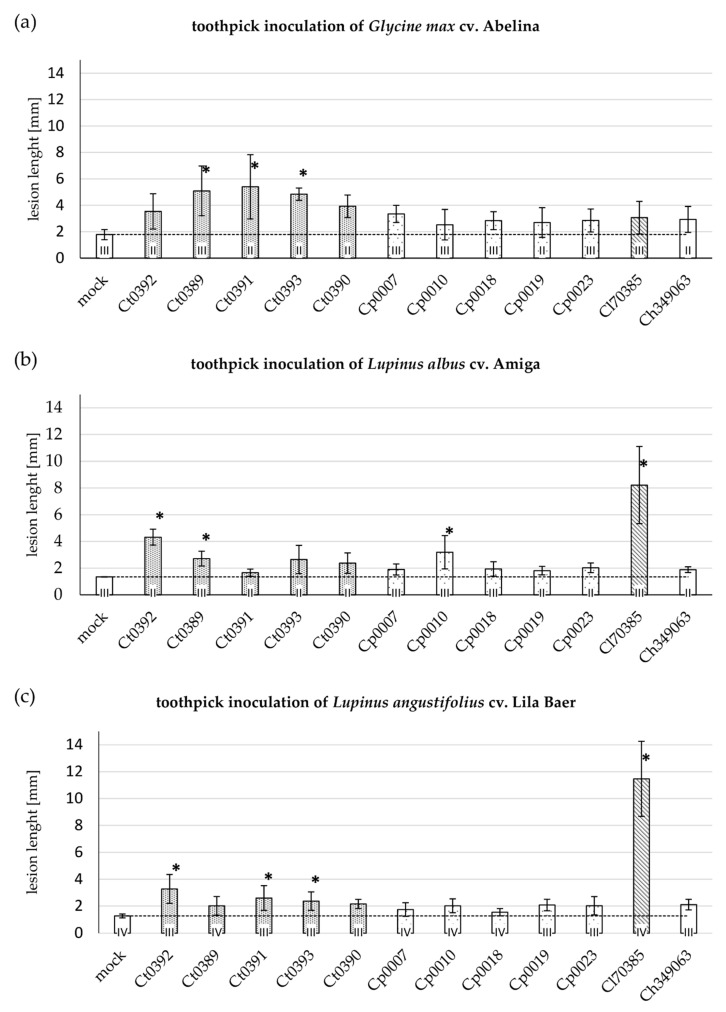
Results of lesion length measurements after inoculation of soybean and lupin hypocotyls. Hypocotyls of *G. max* cv. Abelina (**a**), *L. albus* cv. Amiga (**b**), and *L. angustifolius* cv. Lila Baer (**c**) were infected with *Colletotrichum*-inoculated toothpick tips and lesion lengths were measured after seven days. The abbreviations *Ct*, *Cp*, *Cl*, and *Ch* represent the species *C. truncatum*, *C. plurivorum, C. lupini*, and *C. higginsianum*, respectively. Bars represent the mean value of two to four independent biological experiments with two plants each. The roman numerals represent the number of independent experiments. Error bars show standard deviation. Asterisks indicate a statistically significant difference in comparison with the mock control, determined by a one-way ANOVA analysis on ranks, followed by Dunn’s post hoc test (*p* < 0.05). In the graphs, the dashed line represents the mean value of the mock control.

Some plant pathogenic fungi from the genus *Colletotrichum* are known to have a broad host range and, in addition, their lifestyles can range from necrotrophy to endophytic behavior [2,21]. In this study, we firstly investigated *Colletotrichum* species originating from soybean or lupin for their ability to cross-infect both hosts. The infection process of *Colletotrichum* species on host plants is mediated by a specific infection structure called appressorium. This specialized cell is melanized and facilitates a direct penetration of the host cuticle and cell wall by weakening the plant tissue with the help of secreted lytic enzymes in combination with physical pressure driving the entry of the infection hypha [22,23,24,25]. To assess the principal ability of the *Colletotrichum* isolates under investigation to colonize a host plant irrespective of appressorium formation, we first employed an infection assay based on fungus-inoculated toothpicks that were directly placed into the seedling’s hypocotyls. In case of successful colonization, lesions formed at the site wounding caused by the toothpicks, and lesion length in comparison with the mock-inoculated control served as a measure for virulence (Figure 1a).

Different field isolates of *C. truncatum* caused the largest lesions on hypocotyls of the German soybean cultivar Abelina (maturity group 000). Mean lesion lengths of the *C. truncatum* isolates LFN0389, LFN0391, and LFN0393 were 5.1, 5.4, and 4.8 mm, respectively, and significantly different from the mean lesion length of 1.8 mm caused by the sterile, non-inoculated toothpick tip alone (Figure 1a). Infection with isolates of *C. plurivorum* and *C. lupini*, as well as *C. higginsianum*, also resulted in lesions larger than the control, but the test for statistical significance failed. For the experiments with lupin, two different plant species were chosen: *L. albus* (broad-leafed, white-flowering sweet lupin) and *L. angustifolius* (narrow-leafed, blue-flowering sweet lupin). *L. albus* cv. Amiga was most susceptible to *C. lupini* isolate BBA70358 (mean lesion length: 8.2 mm) (Figure 1b). The soybean-derived *C. truncatum* isolates LFN0392 and LFN0389 were able to cause lesion sizes (mean lesion length of 4.3 and 2.7 mm, respectively), which are significantly larger than the mock control but still not as large as those caused by *C. lupini*. Furthermore, the *C. plurivorum* isolate LFN0010, also derived from soybean, was able to colonize *L. albus* hypocotyls to a similar extent as the *C. truncatum* isolates (Figure 1b). A similar picture was obtained in the toothpick-inoculation experiments with *L. angustifolius* cv. Lila Baer. Again, the adapted *C. lupini* isolate BBA70385 caused the most prominent lesions (mean value of 11.46 mm lesion length, Figure 1c). With mean lesion length ranging from 2.0 to 3.3 mm, the *C. truncatum* isolates LFN0392, LFN0391, and LFN0393 caused smaller, but still significant, lesion lengths.

### 3.2. Cross-Infection Assays Using Inoculated Seeds of Soybean and Lupin

**Figure 2:**
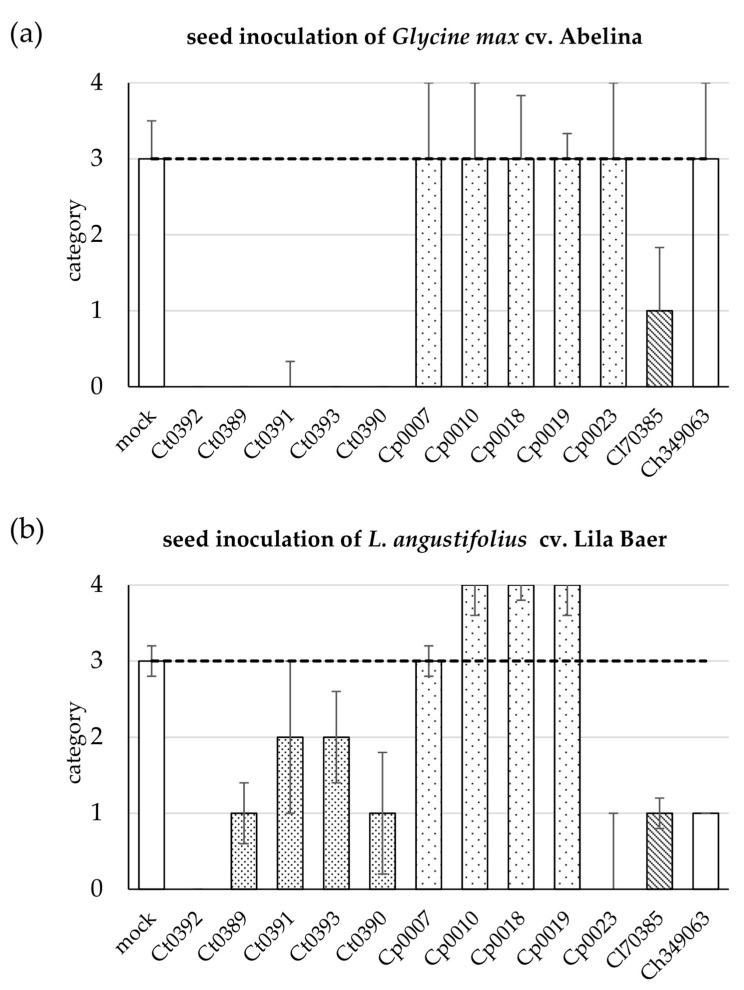
Rating of seedling emergence and development after inoculation of soybean and lupin seeds with *Colletotrichum* isolates. Seeds of *G. max* cv. Abelina (**a**) and *L. angustifolius* cv. Lila Baer plants (**b**) were inoculated with different *Colletotrichum* species and rated one week after sowing. The abbreviations *Ct*, *Cp*, *Cl*, and *Ch* represent the species *C. truncatum*, *C. plurivorum, C. lupini*, and *C. higginsianum*, respectively. The following categories for rating were applied: 0—seedling not emerged; 1—seedling development arrested after emergence; 2—slower or impaired seedling development in comparison with seedlings emerging from mock-inoculated seeds; 3—seedling development like the mock; 4—faster/better development in comparison with mock. Bars represent the median value of five plants and error bars show mean absolute deviation from the median. In both graphs, the dashed line represents the median value of the mock control. The experiment was repeated once with comparable results.

To assess the cross-infectivity of different *Colletotrichum* isolates on soybean and lupin in an independent assay, we performed seed inoculation experiments. This assay is assumed to be closer to the natural mechanism of plant infection because the fungus must infect the seedling without previous wounding. The same set of fungal isolates as in the toothpick-inoculation assay was tested on soybean cv. Abelina and narrow-leafed lupin cv. Lila Baer (Figure 2).

Because not all seeds progressed to normal-looking seedlings, we chose a classification of five categories encompassing a range of non-emerged seedlings (0); seedlings that emerged but did not develop further (1); and seedlings that were smaller (2), equally (3), or better (4) developed in comparison with the mock control. On soybean, all *C. truncatum* isolates had severe effects on seedling emergence, in most cases inhibiting emergence. *C. plurivorum* isolates LFN0007, LFN0010, LFN0018, LFN0019, and LFN0023 caused growth comparable to control, with the tendency of individual plants growing better than the mock-inoculated plants. The *C. lupini* isolate caused retardation of seedling development in comparison with mock-treated seeds one week after sowing. (Figure 2a).

This plant growth-promoting effect of *C. plurivorum* was even more prominent in the seed-inoculation experiment with *L. angustifolius* cv. Lila Baer (Figure 2b). Here, all but one isolate of *C. plurivorum* led to seedlings that developed better than seeds from the non-inoculated control. In this experiment, the *C. plurivorum* isolate LFN0023 led to a similar reduction in seedling development as the *C. lupini* and the *C. higginsianum* isolate. All *C. truncatum* isolates clearly impaired lupin seedling development, and seed inoculation with the *C. truncatum* isolate LFN0392 completely prevented seedling emergence (Figure 2b).

### 3.3. Plant Growth-Promoting Effect on Lupin

**Figure 3:**
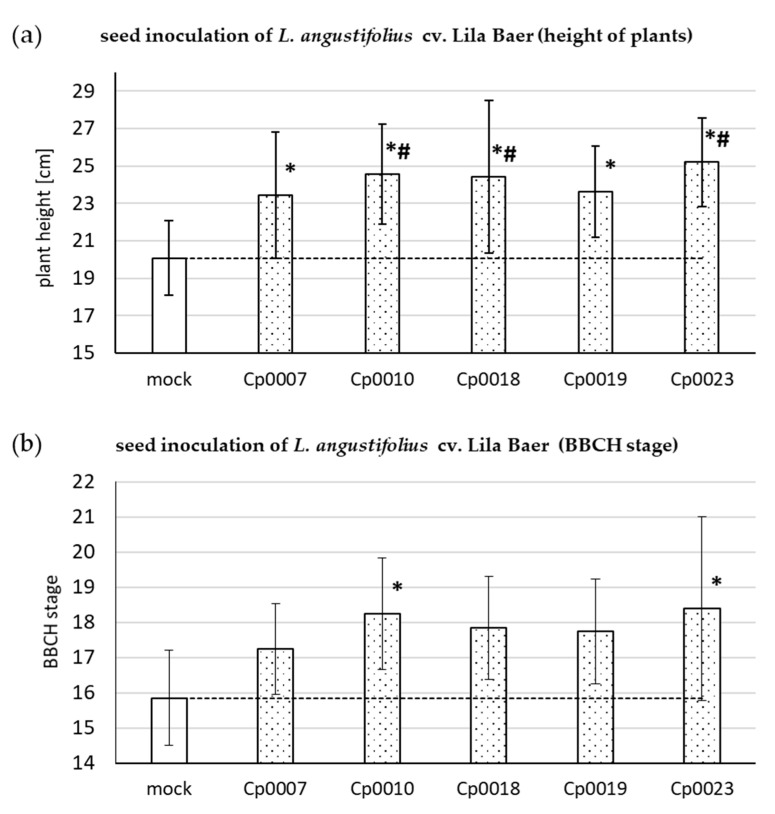
Plant growth-promoting effect of *C. plurivorum* inoculation on lupin development. In an independent experiment, heights of *L. angustifolius* cv. Lila Baer plants after inoculation of seeds with different *C. plurivorum* isolates were measured five weeks after sowing (**a**). The plants were also scored according to a lupin-adapted BBCH development scale (**b**). Bars represent the mean value of at least five plants and error bars show standard deviation in case of (**a**). In (**b**), ordinal data are presented; thus, median and mean absolute deviation from the median are depicted. Statistical significance in relation to the mock inoculation was tested with a one-way ANOVA analysis followed by Dunnett’s (*p* < 0.05, number sign) or Holm-Sidak (*p* < 0.05, asterisk) post hoc test (**a**). In (**b**), the test for statistical significance was performed by a one-way ANOVA analysis on ranks, followed by Dunn’s post hoc test (*p* < 0.05, asterisk). In both graphs, the dashed line represents the mean value of the mock control. The experiment was repeated once with similar results.

The purpose of the seed inoculation experiments was to obtain an overview of the interactions of the whole range of fungal species and isolates in this study; therefore, they were based on single experiments with a lower number of plants. To support the observation regarding the plant growth-promoting effects, an independent biological experiment with *L. angustifolius* cv. Lila Baer and the *C. plurivorum* isolates was performed (Figure 3).

**Figure 4:**
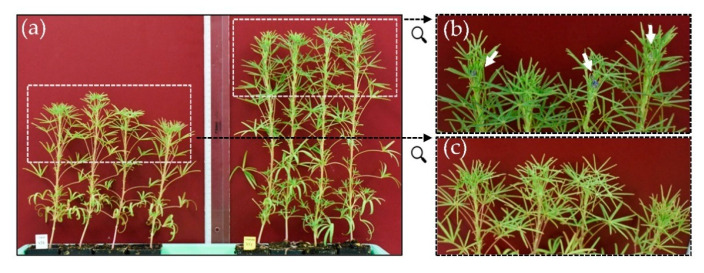
Plant growth-promoting effect of *Colletotrichum plurivorum* in the seed inoculation assay. Comparison of plants from *L. angustifolius* cv. Lila Baer after treatment of seed without (**a**, left side) or with *C. plurivorum* isolate LFN0010 (**a**, right side) seven weeks after sowing. (**b**,**c**) Enlargements of the respective white dashed boxes in (**a**), indicated by black arrows. White arrows in (**b**) point to developing flowers. Pictures correspond to data shown in Figure 3.

Instead of using the previously described rating system, this time, the height of plants was measured, and the developmental stage was assessed according to a lupin-adapted BBCH scale. The plant developed from *C.-plurivorum*-inoculated seeds all were significantly taller than the plants originating from mock-treated seeds after seven weeks (Figure 3a). The rating, according to a lupin-adapted BBCH scale, showed that not only plant height was positively affected, but also the number of leaves (Figure 3b). Whereas the tendency of an increased leaf number could be observed for all interactions with the fungal isolates, only interactions with *C. plurivorum* isolates LFN0010 and LFN0023 proved to be significantly different from the mock control. Figure 4 shows representative plants from this experiment seven weeks after sowing.

The differences between plants of the non-inoculated control and those emerged from seeds treated with the fungus were easily visible (Figure 4a). It seems that seedlings were not only taller after inoculation with *C. plurivorum*; rather, the development as a whole was affected as some of the plants already progressed to the flowering stage (Figure 4b).

### 3.4. Comparison of Potential Soybean and Lupin Cultivation Areas in Germany

**Figure 5:**
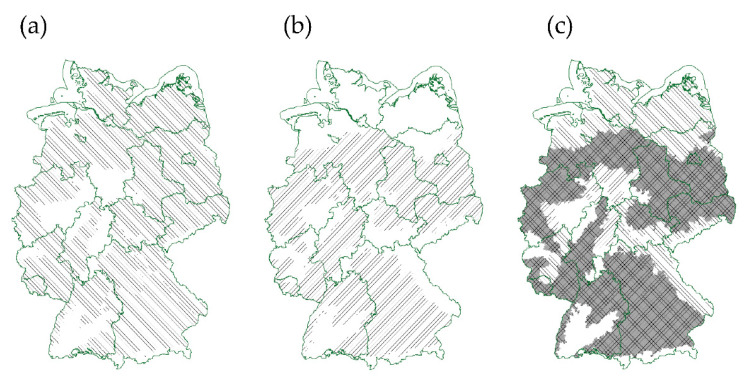
Comparison of potential cultivated area for narrow-leafed sweet lupin and soybean in Germany. Possible areas for cultivation of narrow-leafed sweet lupin (**a** vertical hatching) and soybean (**b**, vertical hatching) based on soil–climate areas are illustrated. An overlay of (**a**) and (**b**) shows the overlap of areas (**c**, gray-underlaid cross-hatching). Data for maps were obtained and modified from Julius Kühn-Institut (geoportal.julius-kuehn.de, accessed 1 May 2021) under license according to German GeoNutzV and from German Federal Agency for Cartography and Geodesy (http://www.bkg.bund.de, accessed 1 May 2021) under Data license Germany—attribution—Version 2.0 (dl-de/by-2-0).

In order to evaluate the potential risk of cross-infection of *Colletotrichum* species originated from soybean or lupin on the other host, we analyzed the suitable production areas in Germany. Data were obtained from Julius Kühn-Institut (http://geoportal.julius-kuehn.de, accessed 1 May 2021) and compiled into a graphic using the free software GRASS GIS (https://grass.osgeo.org/, accessed 1 May 2021). The results showed that potential lupin and soybean cultivation areas overlap in Germany, thus indicating the need for continuous anthracnose monitoring and virulence testing for both crops (Figure 5c).

## 4. Discussion

In this study, the interaction of *Colletotrichum spec.* isolates originating from soybean or lupin was tested for the first time on soybean and lupin cultivars used in Germany. Since, to the best of our knowledge, soybean anthracnose has not yet been systematically investigated on soybean in Germany, we used isolates of *C. truncatum* and *C. plurivorum* from Brazil [7,8].

To obtain an overview of the virulence spectrum of the different fungal isolates on both host plants, at first, an assay with a simple read-out, namely measurement of lesion lengths, was performed. Using toothpicks colonized by fungi as an inoculum source was already established and successfully applied for studying *Fusarium virguliforme*, *C. truncatum,* and *C. plurivorum* on soybean and applied here for the first time to lupin hypocotyls [7,8,18]. This assay is robust and the read-out in the form of lesion length measurement is easily quantifiable. However, the inoculation method included wounding and is thus not closely related to the natural infection process. By wounding, the pre-formed resistance barriers of the cuticle and an intact epidermis are circumvented. Differences in colonization, therefore, must be attributed to defense mechanisms active during the colonization of the plant in response to the invading fungus [18,26]. On soybean cv. Abelina, all *C. truncatum* colonized the hypocotyl beyond the wound-induced lesion, but only the isolates LFN0389, LFN0391, and LFN0393 lead to significantly larger lesions (Figure 1a). The soybean-derived isolates of *C. plurivorum* and *C. lupini* caused smaller lesions than the *C. truncatum* isolates. Remarkably, *C. higginsianum*, known to naturally infect plants from the *Brassicaceae* family, was also able to invade the tissue to a certain extent, albeit not statistically significantly (Figure 1a). This rather unexpected result might be related to the artificial inoculation assay or may also be explained by the exceptional large host range of many species of the genus *Colletotrichum*, which possesses the general ability to grow on a variety of plants as well as dying and dead tissue [21,27].

In the toothpick assay, *C. lupini* caused the largest lesions on both lupin cultivars Amiga and Lila Baer (Figure 1b,c). The interactions of lupin with the other *Colletotrichum* isolates revealed a more differentiated picture between *L. albus* (Figure 1b) and *L. angustifolius* (Figure 1c), which might be attributed to differences in the basal resistance of both lupin cultivars.

Since anthracnose of soybean and lupin is reported to be seed-borne, we decided to also employ a seed infection assay to more accurately mimic the natural infection process [2,11]. For this assay, seeds were co-incubated with the fungus on agar plates. The swelling of the seeds followed by germination, which quickly happens after placing seeds on agar-medium, made this method inadequate. We prevented swelling and germination by increasing the osmotic potential of the agar medium, enabling a longer co-incubation phase for seed and fungus, resulting in evenly inoculated seeds [18]. Soybean seeds pre-treated this way with *C. truncatum* isolates only rarely developed seedlings, and again, *C. lupini* and *C. higginsianum* showed a tendency to cause retarded seedling development (Figure 2a). In the case of *L. angustifolius* cv. Lila Baer, the *C. truncatum* isolate LFN0392 exhibited a stronger negative effect on seedling emergence than *C. lupini* (Figure 2b). Most interestingly, in addition to causing retardation of seedling development, some *C. plurivorum* interactions led to promotion of plant growth of both soybean and lupin seedlings (Figure 2). These findings were confirmed in an independent experiment using *L. angustifolius* and the *C. plurivorum* isolates (Figure 3). In addition to evaluation in the first experiment, the development of plants was monitored over a longer time span. After five weeks, the plant growth-promoting effect could be observed for all interactions tested. This was not only manifested by increased plant height (Figure 3a), but also by the advancement to more mature developmental stages as observed in comparison with plants growing from mock-treated seeds (Figure 3b). Thus, after seven weeks, some of the inoculated plants already progressed to the flowering stage (Figure 4).

In contrast with the first experiment, the isolate LFN0023 also caused a plant growth-promoting effect. Our results underline the plasticity of the interaction of *Colletotrichum* spp. with different hosts, which can range from pathogenicity to commensalism and mutualism. Maybe also external factors influence this balance, as a substrate poor in nutrients was used in the second experiment and plants were grown longer than in the first experiment. It is known that *Colletotrichum* species exhibit a broad spectrum of different lifestyles ranging from endophytic growth to necrotrophic behavior, and that dynamic transitions in lifestyle can happen depending on host plants and environmental factors [21,28,29]. 

Our study revealed that soybean-derived isolates of *Colletotrichum* spp. are capable of causing disease on lupin, and that *C. lupini*, at least under laboratory conditions, can colonize soybean. Whether this may cause problems in legume production in Germany cannot be foreseen, but considering the overlap of areas where soybean and lupin production is possible, this might only be a matter of time (Figure 5).

In this study, we reported a plant growth-promoting effect of fungi from the genus *Colletotrichum* for legumes; however, the underlying mechanism remains enigmatic. Future studies must explore the mechanism and should address the question as to whether the fungus can grow endophytically such as, e.g., *Piriformospora indica* (syn. *Serendipita indica* [30,31]. It will be interesting to see whether this beneficial interaction might render plants more resistant to abiotic and biotic stresses. Ultimately these results can contribute to novel approaches in legume production.

## Figures and Tables

**Table 1:** Fungal isolates used in this study.

Species	Strain	ID in Manuscript	Origin	Literature
*Colletotrichum lupini*	BBA70358 (= CBS109222)	Cl70358	lupin	[12,14]
*C. truncatum*	LFN0389	Ct0389	soybean	[8]
*C. truncatum*	LFN0390	Ct0390	soybean	[8]
*C. truncatum*	LFN0391	Ct0391	soybean	[8]
*C. truncatum*	LFN0392	Ct0392	soybean	[8]
*C. truncatum*	LFN0393	Ct0393	soybean	[8]
*C. plurivorum*	LFN0007	Cp0007	soybean	[8,10]
*C. plurivorum*	LFN0008	Cp0008	soybean	[8,9,10,16]
*C. plurivorum*	LFN0010 (= URM7540)	Cp0010	soybean	[7,8,16]
*C. plurivorum*	LFN0018 (= URM7541)	Cp0018	soybean	[7,8,16]
*C. plurivorum*	LFN0019 (= URM7542)	Cp0019	soybean	[7,8,16]
*C. plurivorum*	LFN0023	Cp0023	soybean	[16]
*C. higginsianum*	IMI349063	Ch349063	Arabidopsis	[17]

## Data Availability

The data presented in this study are available on request from the corresponding author.
